# Genomic Strategies for Understanding the Pathophysiology of Autism Spectrum Disorder

**DOI:** 10.3389/fnmol.2022.930941

**Published:** 2022-06-24

**Authors:** Miyuki Doi, Mengwei Li, Noriyoshi Usui, Shoichi Shimada

**Affiliations:** ^1^Department of Neuroscience and Cell Biology, Graduate School of Medicine, Osaka University, Suita, Japan; ^2^Addiction Research Unit, Osaka Psychiatric Research Center, Osaka Psychiatric Medical Center, Osaka, Japan; ^3^United Graduate School of Child Development, Osaka University, Suita, Japan; ^4^Global Center for Medical Engineering and Informatics, Osaka University, Suita, Japan

**Keywords:** genomics, autism spectrum disorder (ASD), gene, mutation, cellular model, animal model, morphology, behavior

## Abstract

Recent breakthroughs in sequencing technology and technological developments have made it easier to analyze the entire human genome than ever before. In addition to disease-specific genetic mutations and chromosomal aberrations, epigenetic alterations in individuals can also be analyzed using genomics. Autism spectrum disorder (ASD) is a neurodevelopmental disorder (NDD) caused by genetic and/or environmental factors. More than a thousand genes associated with ASD have been identified which are known to be involved in brain development. However, it is difficult to decode the roles of ASD-associated genes without *in vitro* and *in vivo* validations, particularly in the process of brain development. In this review, we discuss genomic strategies for understanding the pathological mechanisms underlying ASD. For this purpose, we discuss ASD-associated genes and their functions, as well as analytical strategies and their strengths and weaknesses in cellular and animal models from a basic research perspective.

## Introduction

ASD is an NDD caused by genetic and/or environmental factors, as reported in many epidemiological studies. ASD symptoms are heterogeneous and are characterized by social communication deficits, repetitive behaviors, and hyperesthesia/hypesthesia (Lord et al., 2018, 2020). The number of ASD patients is increasing worldwide. In the latest report, the prevalence of ASD was reported to be 1 in 44 (2.27%) in the US (Maenner et al., 2021; Shaw et al., 2021). Nevertheless, no effective treatment has been developed to date. Although early intervention for ASD has been reported to improve symptoms (White et al., 2007; Dawson et al., 2012; Rogers et al., 2012), this depends on symptomatic treatment such as cognitive behavioral therapy. Therefore, it is necessary to develop early treatment and diagnostic methods as soon as possible. To understand the pathophysiology underlying ASD, identification of causative genes in individuals and environmental factors during fetal development, and research and development using cellular and animal models, have been undertaken.

Genetic and/or environmental factors contribute to the pathogenesis of ASD (Lord et al., 2018, 2020; Doi et al., 2022). Massive genome-wide association studies (GWASs) in ASD have been conducted using advanced next-generation sequencing (NGS) technology. Over a thousand ASD-associated genes have been identified in individuals with ASD, suggesting that ASD is a highly complex disorder (Iossifov et al., 2014; Toma et al., 2014; Griswold et al., 2015; Wilkinson et al., 2015; Yao et al., 2015; Satterstrom et al., 2020; Wang et al., 2020). Most ASD genes are known to play roles in fetal brain development, particularly in neurogenesis, neuronal migration, neural differentiation, and synaptogenesis (Tebbenkamp et al., 2014; Wang et al., 2014; de la Torre-Ubieta et al., 2016; Doi et al., 2022). Furthermore, environmental factors, such as maternal immune activation (MIA), stress, undernutrition, and drug exposure have also been investigated to understand the pathophysiology of ASD (Beversdorf et al., 2005; Christensen et al., 2013; Gumusoglu and Stevens, 2019; Meyer, 2019; Courchesne et al., 2020; Doi et al., 2022).

To investigate the involvement and functions of these ASD genes, there are several strategies that use cellular and animal models instead of humans. For cellular models, morphological and electrophysiological phenotypes in cells can be evaluated by manipulating the expression of ASD genes using cell culture systems (Gordon and Geschwind, 2020). In recent years, studies using organoids cultured in three dimensions, in addition to neural cells and intermediate progenitor cells, have been conducted (Kelley and Paşca, 2022). As for animal models, various animal species such as mice, rats, macaques, zebrafish, fruit flies and nematodes are widely used, often taking advantage of powerful genome editing technology. The strength of animal models is their ability to analyze gene function at the individual gene level. In particular, neural circuit and behavioral analyses are the most important features of animal models (Silverman et al., 2010; Kazdoba et al., 2016a).

Here, we review the genomic strategies for understanding the pathophysiology of ASD. In this review, we first discuss how ASD genes have been identified and the brain functions to which they have been traced. We will also review strategies for understanding the pathophysiology of ASD, focusing on cellular and animal models and their advantages and disadvantages, and introduce the latest research findings and strategies, including our own.

## Exploration and Identification of ASD-Associated Genes

Among the genetic studies, twin studies have contributed to the identification of factors involved in the pathogenesis of ASD. Since the first twin study on ASD in 1977, which reported a higher concordance in monozygotic (MZ) twins than in dizygotic (DZ) twins for ASD (Folstein and Rutter, 1977), many studies have applied twin designs (Ronald and Hoekstra, 2011; Sandin et al., 2014). Furthermore, a meta-analysis of twin studies conducted in 2015 estimated a heritability of 64–91% (Tick et al., 2016). Based on these studies, it is widely accepted that genetic factors play a crucial role in ASD pathogenesis. Some genetic disorders consistently linked to ASD, including Angelman syndrome, fragile X syndrome, tuberous sclerosis, Rett syndrome, and other *MECP*2-related disorders, Smith-Lemli-Opitz syndrome, phenylketonuria, Cohen syndrome, Smith-Magenis syndrome, Sanfilippo syndrome, adenylosuccinate lyase deficiency, Down syndrome, 22q13 deletion syndrome (Cohen et al., 2005; Bukelis et al., 2007).

Progress in genetics over the past few decades has provided insights into the genomic and molecular mechanisms underlying ASD. Earlier studies using microarray and next-generation sequencing, such as whole exome sequencing (WES) and whole genome sequencing (WGS), have identified contributions from both rare (e.g., *de novo* and inherited mutations) and common variants [e.g., copy number variants (CNVs) and single nucleotide variants (SNVs); State and Levitt, 2011; de la Torre-Ubieta et al., 2016; Muhle et al., 2018]. Unlike inherited mutations, which are passed from the parent, *de novo* mutations are spontaneous “novel” mutations that arise in gametes or fertilized eggs, and cannot be identified in the parent. CNVs are chromosomal abnormalities which include deletions, duplications, or amplifications in chromosomal structure; it has been found that there are multiple chromosomal abnormalities associated with ASD recurring at specific loci (Sanders et al., 2011). Sanders et al. also reported two or more recurrent deletions and duplications at multiple loci, such as deletions at 16p11.2 and duplications at 15q13.2–13.3 in patients with ASD (Sanders et al., 2011).

Although common variants are estimated to have a more substantial impact than rare variants on ASD liability (Klei et al., 2012; Lee et al., 2013; Gaugler et al., 2014), many genome-wide association studies (GWAS) have been insufficient to identify even a single SNV with genome-wide significance until recently, owing to the massive number of variants involved as well as the low risk associated with ASD individually (Wang et al., 2009; Anney et al., 2010, 2012; Autism Spectrum Disorders Working Group of The Psychiatric Genomics Consortium, 2017). In other words, investigating the influence of single SNVs requires a large sample number. However, a study published in 2019, employing a huge cohort and strict analysis, identified 34 high-confidence ASD (hcASD) risk genes at multiple genetic loci (Grove et al., 2019). They also reported that these ASD-candidate genes are more highly expressed during the embryonic stage than during the postnatal stages, suggesting their roles in fetal brain development (Courchesne et al., 2019).

**Figure 1:**
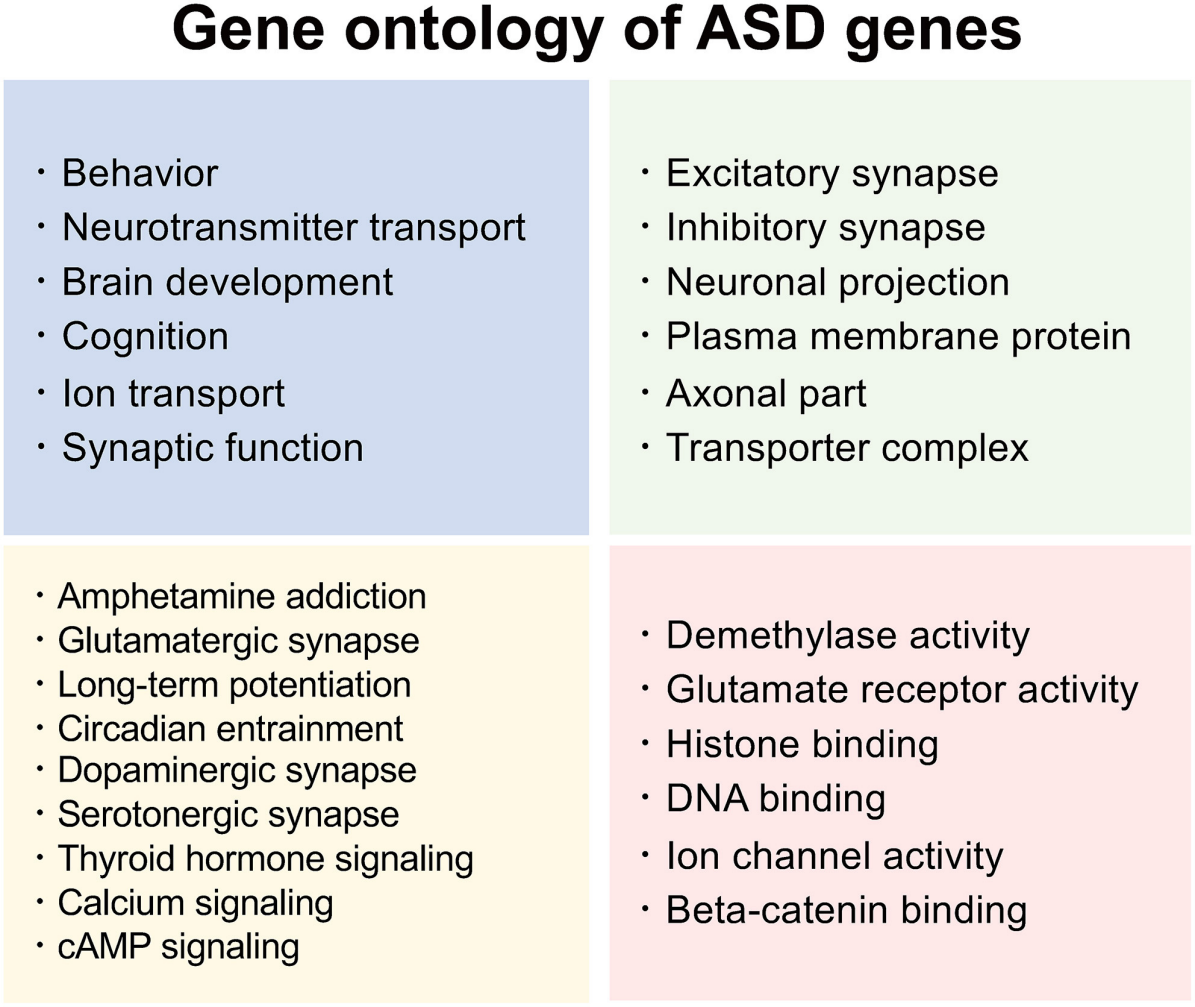
Summarized functions of ASD genes. Functional and related pathway information based on gene ontology of the latest SFARI ASD genes (1,031 genes released on January 11, 2022). SFARI Gene: https://www.sfari.org/resource/sfari-gene.

Considering the significance of heritability and intricate genetic factors in ASD, a thorough database of known ASD-associated genes would be invaluable. SFARI gene (https://gene.sfari.org/) is a well-known database of ASD-associated genes (Banerjee-Basu and Packer, 2010) which is widely used in ASD research. In SFARI, each gene is categorized into one of four groups: S (syndromic), 1 (high confidence), 2 (strong candidate), and 3 (suggestive evidence). In addition, SFARI gene provides functional information on ASD genes, making it an indispensable tool for investigating the effect of a gene during development. For example, one of the frequently reported ASD genes, *SHANK3*, is a multidomain scaffold protein involved in the regulation of the structural organization of dendritic spines (Durand et al., 2007). Another frequently reported ASD-associated gene, *MECP2*, plays a crucial role in chromatin modification and RNA silencing (Li and Pozzo-Miller, 2020). *CNTNAP2* modulates cell adhesion, dendritic arborization, and spinogenesis (Li and Pozzo-Miller, 2020). *FOXP1* is also frequently reported to be involved in social communication, cortical development, spatial learning, synaptic plasticity, and the regulation of ASD-associated gene expression (Araujo et al., 2015, 2017; Usui et al., 2017a). *FOXP2* regulates vocal communication, cortical development, behavioral flexibility, and ASD-associated gene expression (Usui et al., 2014, 2017b; Co et al., 2020). Collectively, predicting gene ontology of the SFARI genes implicates the majority of them in brain development (Figure 1), with most of them being synapse-associated genes (de la Torre-Ubieta et al., 2016).

The expanding knowledge of ASD genes and their functions offers further insights into ASD pathogenesis. Efforts have been made in epigenomics for many identified ASD-associated genes involved in epigenetic pathways, such as *MECP2*. A significant reduction in *MECP2* in the frontal cortex of individuals with ASD has been reported, suggesting that disruption of DNA methylation plays a role in ASD pathogenesis (Nagarajan et al., 2006). Other epigenomic studies have found similar aberrations in histone acetylation (Sun et al., 2016), correlating with DNA methylation variations in the prefrontal and temporal cortices (Wong et al., 2019). In addition to postmortem brain samples and blood samples have also been used in ASD-associated DNA methylation examination (Andrews et al., 2018), suggesting a potentially more convenient approach for DNA methylation studies of ASD. It seems that epigenomics might be ideal for finding patterns of convergence among heterogeneous ASD-associated genes and addressing the complex effects resulting from the interaction of genomics and the environment.

Together, studies on the genetic contributions to ASD pathogenesis are crucial to elucidate ASD etiology and fetal brain development. These genomic studies also provide insights into the heterogeneity of ASD, which is thought to contribute to the complexity of ASD pathogenesis.

## Cellular Models for Understanding ASD Pathophysiology

The utility of cellular models in ASD studies is that human cells such as patient-derived induced pluripotent stem cells (iPSCs) can be used to avoid ethical issues (Bellin et al., 2012). It is also more feasible to study a relatively simple system rather than a complicated one in the living body. In addition, cellular models often require less time and space than animal models. It is expected that the development of ASD modeling at the cellular level will promote at least a partial understanding of the functional and morphological characteristics underlying ASD pathophysiology (Gordon and Geschwind, 2020). Furthermore, cellular models that reflect pathological conditions can be helpful tools for drug development; therefore, versatile cellular models are attracting increasing attention.

There are multiple options for the selection of cellular models. The first method is to use cells derived directly from individuals with ASD. Patient-derived iPSCs are a powerful tool for understanding ASD pathophysiology. A method for introducing mutations identified in patients with ASD into iPSCs is also used. Another option is to use genetic recombination or gene-editing techniques to uncover the function of ASD-associated genes using primary cultured neural cells or cell lines. For example, Marchetto et al. (2017) reprogrammed fibroblasts to generate iPSCs, neural progenitor cells (NPCs), and neurons from ASD individuals, and found that abnormal neurogenesis and synaptogenesis resulted in functional defects in neural networks in ASD. Deneault et al. selected ASD-associated genes based on their WES results and generated knockout (KO) iPSCs using CRISPR/Cas9 genome editing. They classified cells based on their transcriptome data and found that similar electrophysiological phenotypes can be produced by ASD-associated genes from different classifications (Deneault et al., 2018). Hashimoto et al. (2016) identified ASD-associated genes with *de novo* SNVs using trio-based WES in the ASD family, and generated knockdown neuroblastoma cells to investigate their morphological characteristics.

It is well-known that both neurons and glia (astrocytes, oligodendrocytes, and microglia) contribute to the pathophysiology of ASD. Knockdown of the ASD-associated gene *NRXN* using shRNA in NPCs resulted in inhibition of astrogenesis. Furthermore, astrocytes generated from iPSC derived from ASD individuals with *NRXN1* deletions show abnormal morphology and proliferation (Lam et al., 2019). In another study, Allen et al. (2022) transplanted astrocytes generated from ASD individual-derived organoids into the mouse brain and observed repetitive behavior, cognitive deficits, and abnormal neuronal activity in the transplanted mice. In addition, aberrant myelination has been reported in ASD postmortem brain (Fetit et al., 2021). Such myelin is formed by oligodendrocytes. In the study of ASD-associated gene *ZBTB16*, the reduced numbers of oligodendrocytes as well as impairment of cortical myelination have been reported in *Zbtb16* KO mice, which displayed ASD-like behaviors (Usui et al., 2021). Increased numbers of synapses have been reported in ASD postmortem brain (Tang et al., 2014; de la Torre-Ubieta et al., 2016). Since microglia play a role in synaptic pruning, microglial dysfunction can disrupt the synaptic excitatory and inhibitory balance (E/I balance). E/I imbalance is one of the explanations for the pathophysiology of ASD (Koyama and Ikegaya, 2015). Moreover, an increasing number of activated microglia and abnormal morphological changes have been reported in the frontal lobes and cerebellum of ASD individuals (Andoh et al., 2019).

As described above, human cells can be used in cellular models, and offer an advantage that animal models do not. In recent years, it has become possible to construct tissues by three-dimensional culture, such as organoids; research is being conducted to mimic the development of the human brain and analyze the pathophysiological conditions of the mimicked environment.

## Animal Models for Understanding ASD Pathophysiology

In contrast to cellular models, animal models allow one to evaluate individuality, such as behavioral and developmental characteristics. Meanwhile, animal ethics issues, breeding environments, and controls are required, which are space-consuming and costly. It also takes a longer time to generate a model to be used in experiments than cellular models. There are various methods (e.g., KO, knock-in, point mutation, and overexpression) to generate ASD animal models in various species. As a typical example, animal models of ASD are generated by modifying the expression of ASD-associated genes or introducing mutations identified in patients with ASD. Various animal species, such as mice, rats, macaques, zebrafish, fruit flies and nematodes have been used as ASD animal models (Stewart et al., 2014; Escamilla et al., 2017; Zhou et al., 2019; Li et al., 2021; Watanabe et al., 2021; Silverman et al., 2022). Therefore, researchers can select animal models based on their purpose. For example, it has reported *Shank2* KO rats as an ASD model animal and repetitive behaviors as well as hyper activity (Arroyo-Araujo et al., 2019). Zhou et al. (2019) introduced a mutation in the macaque *SHANK3* gene. The *SHANK3* mutant macaques showed a reduction of its protein level and ASD-like behaviors such as social impairment and repetitive behaviors. The *SHANK3* mutant model has been generated in zebrafish. In *shank3a* knockdown zebrafish displayed a decrease of head size and impairment of touch-induced startle response (Stewart et al., 2014).

To assess behavioral phenotypes using animal models, ASD-like behaviors can be evaluated in terms of social and restricted/repetitive behaviors. Social behaviors can be assessed by the three-chamber social interaction, free moving social interaction, social preference, ultrasonic vocalization tests (Silverman et al., 2010; Lammert and Lukens, 2019). Restricted/repetitive behaviors can also be assessed by stereotyped behaviors, such as grooming, rearing, climbing behaviors, and marble burying test (Silverman et al., 2010; Langen et al., 2011; Lammert and Lukens, 2019). For example, a mutation in the transcription factor *ZBTB16* was identified in brothers with ASD (Bacchelli et al., 2019). Recently, we reported impairments in social behaviors and increased repetitive behaviors in *Zbtb16* KO mice (Usui et al., 2021). Furthermore, histological abnormalities in cortical layer thickness, axon initial segment (AIS) length of neurons, and myelination in the cortex have been reported in *Zbtb16* KO mice (Usui et al., 2021, 2022b). *SHANK3, NLGN4*, and *NRXN1* are involved in synaptogenesis. *CADPS2*, implicated in the transport and secretion of dense-core vesicles, has been identified as an ASD-associated gene, and its KO mice exhibit ASD-like behaviors (Jamain et al., 2008; Peça et al., 2011; Grayton et al., 2013).

Animal models are also useful for analyzing environmental factors. Previous genomic studies demonstrate that about 20–30% of patients with ASD have abnormal genes (Kazdoba et al., 2016b), suggesting the importance of environmental factors in the onset of ASD. MIA and drug exposure animal models have also been used (Doi et al., 2022; Usui et al., 2022a).

Thus, animal models have advantages over cellular models. In recent years, studies have been conducted from the perspective of systems neuroscience using two-photon microscopy, multi-electrode arrays, and optogenetics, etc., in addition to genomics. Previous studies using these methods have begun to unravel the neural circuits and mechanisms responsible for social behavior, which is one of the main symptoms of ASD.

## Discussion

Analyses of the functions of ASD-associated genes have revealed that most of these genes are involved in fetal brain development. In recent years, provocative studies have been conducted to examine whether ASD can be treated by genome editing during the fetal period, when the brain is dynamically developed. However, given the fact that approximately half of ASD cases can be attributed to genetic factors (Sandin et al., 2014), environmental factors also appear to contribute to the pathogenesis of ASD. Environmental factors such as MIA and drug exposure are known to affect fetal brain development in the prenatal environment (Doi et al., 2022). However, some environmental factors, such as MIA due to infectious diseases, could be more difficult to prevent than genetic factors.

ASD is a heterogeneous neurodevelopmental disorder, and such complexity is largely due to the complexity of the onset factors and is difficult to explain using a simple model. ASD is far more complicated than a single-gene disorder, and has a pathogenesis that is due to the accumulation of many predisposing genes, even if only genetic factors are considered. Furthermore, as suggested by the male-female ratio of ASD prevalence, sex differences further add complexity to the symptoms and pathogenesis of ASD. Future studies on the pathological mechanisms underlying ASD that consider both genetic and environmental factors are required.

**Figure 2:**
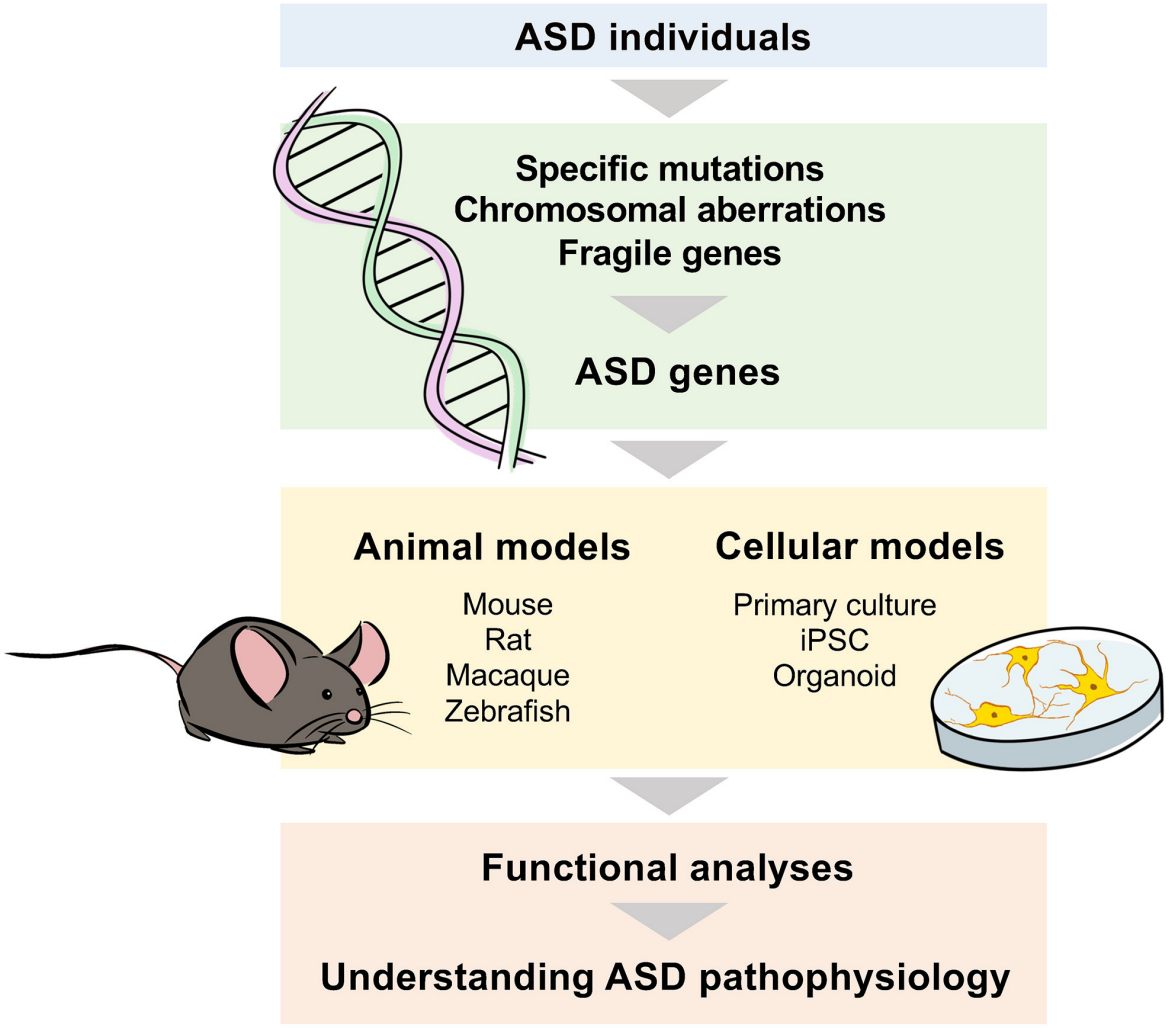
Genomic approaches to understand the mechanisms underlying ASD pathophysiology. To identify the genes involved in the pathophysiology of ASD, genome or gene expression analyses with samples such as blood and postmortem brains from individuals with ASD are performed to identify disease-specific mutations, chromosomal aberrations, and fragile genes. Cellular and animal models are used to analyze the genes identified in ASD individuals, and functional analyses are conducted based on the strength of each model.

In conclusion, genomics is a powerful strategy to understand the pathological mechanisms underlying ASD (Figure 2). The use of animal models is indispensable for understanding complex pathological conditions, but incorporating human findings and pathological conditions will also be essential.

## Author Contributions

MD: writing—original draft, writing—review and editing, and visualization. ML: writing—original draft and visualization. NU: conceptualization, writing—original draft, writing—review and editing, project administration, and funding acquisition. SS: writing—review and editing, supervision, and funding acquisition. All authors have contributed to the manuscript and approved the submitted version.

## Funding

This work was supported by the Japan Science and Technology Agency (JST) Center of Innovation Program (COI Program) (JPMJCE1310) to NU and SS; Japan Society for the Promotion of Science (JSPS) Grant-in-Aid for Scientific Research (C) (20K06872) to NU; JSPS Grant-in-Aid for Challenging Research (20K21654) to NU and SS; Uehara Memorial Foundation to NU; Takeda Science Foundation to NU; SENSHIN Medical Research Foundation to NU; Osaka Medical Research Foundation for Intractable Diseases to NU.

## Conflict of Interest

The authors declare that the research was conducted in the absence of any commercial or financial relationships that could be construed as a potential conflict of interest.

## Publisher's Note

All claims expressed in this article are solely those of the authors and do not necessarily represent those of their affiliated organizations, or those of the publisher, the editors and the reviewers. Any product that may be evaluated in this article, or claim that may be made by its manufacturer, is not guaranteed or endorsed by the publisher.
